# Determination of the Antimicrobial Effects of Synbiotic Kefir Produced from Buffalo Milk Enriched with Galactooligosaccharides and Inulin

**DOI:** 10.1021/acsomega.5c10532

**Published:** 2026-01-22

**Authors:** Aysel GÜLBANDILAR, Neslihan ÇALIŞIR, Muhammet İrfan AKSU

**Affiliations:** † Eskişehir Osmangazi University, Faculty of Agriculture, Department of Food Engineering, 26160 Eskişehir, Türkiye; ‡ Atatürk University, Faculty of Agriculture, Department of Food Engineering, 25240 Erzurum, Türkiye

## Abstract

In this study, the effects of fat content (0.5 and 3.5%), prebiotics (galactooligosaccharides [GOS] and inulin), production method (traditional and commercial), and storage period (21 days at 3 ± 1 °C) on the antimicrobial activity of synbiotic kefirs produced using buffalo milk against certain pathogenic microorganisms (*Staphylococcus aureus*, *Bacillus subtilis*, *Enterococcus faecalis*, *Listeria monocytogenes*, *Pseudomonas aeruginosa*, *Escherichia coli*, and *Candida albicans*) were determined. Variations in fat content significantly affected antimicrobial activity against *E. faecalis* (*p* < 0.01), *L. monocytogenes* (*p* < 0.01), and *E. coli* (*p* < 0.01); the production method influenced *S. aureus* (*p* < 0.01), *B. subtilis* (*p* < 0.05), *E. faecalis* (*p* < 0.01), *E. coli* (*p* < 0.01), and *C. albicans* (*p* < 0.01), while prebiotic addition affected *S. aureus* (*p* < 0.01), *L. monocytogenes* (*p* < 0.05), *E. coli* (*p* < 0.05), and *C. albicans* (*p* < 0.01). The storage period had a significant effect (*p* < 0.01) on all examined pathogens, and antimicrobial activity increased as storage time progressed. In general, the use of commercial culture and prebiotics in kefir production enhanced antimicrobial activity. The effects of inulin (on *S. aureus*, *C. albicans*) and GOS (on *L. monocytogenes*, *E. coli*, *C. albicans*) varied across microorganisms. Based on the average zone diameters produced by the kefir samples: The highest activity against *S. aureus* was observed in inulin-containing and cultured samples, against *B. subtilis*, it was observed on the seventh day in cultured samples, against *C. albicans*, in inulin-containing and cultured samples (on day 21), against *L. monocytogenes*, in GOS-enriched and 0.5% fat samples, as well as in inulin-added samples (on day 21), against *E. faecalis*, in 0.5% fat and cultured samples (on day 21), against *E. coli*, in 3.5% fat, GOS-added, and cultured samples, and against *P. aeruginosa*, activity was detected on the seventh and 14^th^ days of storage.

## Graphical abstract

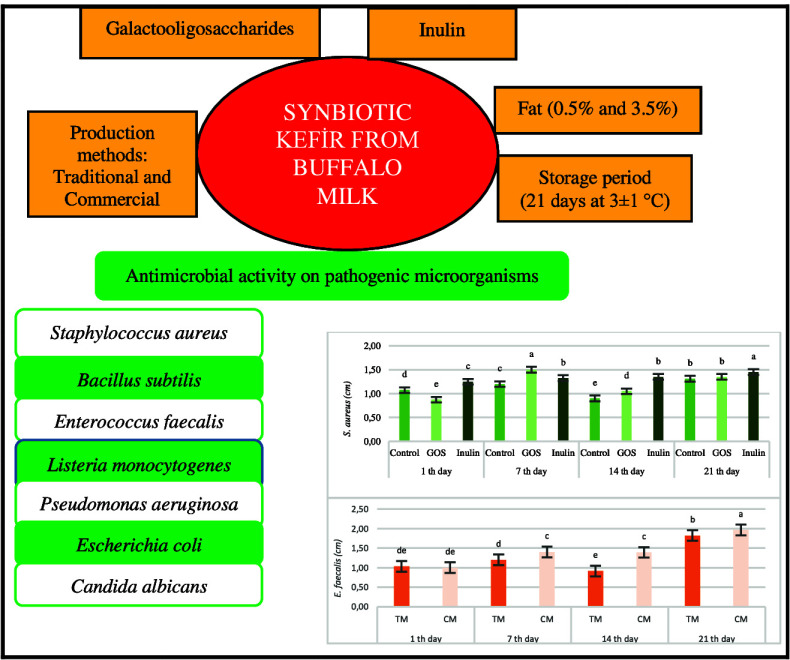

## 1 Introduction

Antimicrobial resistance is a global public health issue. Many bacteria that cause serious infections and were once successfully treated with several different classes of antibiotics have now developed resistance to many of them.^1^ The increasing resistance of pathogenic microorganisms is generally attributed to the improper use of antibiotics and the transmission of resistance within and between individuals. It has been reported that the production of new antibiotics in the industry does not attract the interest of investors and is considered not to be cost-effective. Therefore, it is emphasized that new strategies are needed to prevent the emergence and spread of drug resistance, inhibit bacterial growth, and prolong the effectiveness of conventional antibiotics.^2^ In this context, the natural structures of food raw materials and the transfer and effects of bioactive compounds with antimicrobial properties found in their natural compositions are highly significant. Thus, identifying the antimicrobial properties of foods has become increasingly important in determining or enhancing their functional characteristics.^3–5^ Today, researchers are focusing on the importance of naturally derived bioactive compounds (BACs), which are secondary metabolites obtained from seeds, foods, and fermentation-based metabolic products. Therefore, the isolation of such natural BACs is considered to be promising for multifunctional extracts that can be used in food applications to support health-promoting effects in host cell systems.^6^ It has been reported that in synbiotic foods, particularly those with enhanced probiotic properties, the antimicrobial effect of probiotics may also result from the coaggregation of different types of cells that bind pathogens into aggregates, thereby preventing the growth and biofilm formation of pathogens. Additionally, this coaggregation may prevent pathogen colonization through the formation of a cellular barrier.^7^ In this context, fermented dairy products are attracting attention, and kefir is one of these products. Kefir is a fermented dairy product that is a source of protein, health-promoting bacteria, and carbohydrates. It is all functional attributes arise due to fermentation; therefore, it is important to highlight that it is a fermented milk product. These functional properties of kefir arise from its probiotic microorganisms and the bioactive compounds formed through microbial metabolism.^8–10^ The probiotic microorganisms present in kefir confer antibacterial and anti-inflammatory properties to the product, making it functional.^11^ Kefir grains play the most significant role in acquiring these properties. These grains have a complex microbiota,^12^ and their microbial profile includes *Lactobacillus*, *Lactococcus*, *Leuconostoc*, *Streptococcus*, *L. kefiri*, *L. kefiranofaciens*, *L. kefirgranum*, *L. parakefir*, acetic acid bacteria, and yeasts.^13^ They are composed of a complex symbiotic microbial ecosystem of bacteria and yeasts embedded in an exopolysaccharide matrix made of d-glucose and D-galactose (glucogalactan). These grains account for a significant portion of kefir’s dry weight, around 24–25%.^7^

Fermented milk with kefir grains can be used, especially in the prevention of certain infections related to the gastrointestinal system. The antibacterial properties of kefir are associated with a combination of various factors, including competition for nutrients and the natural effects of organic acids, H_2_O_2_, acetaldehyde, CO_2_, and bacteriocins produced during the fermentation process. These substances are also shown to exhibit effects similar to those of nutraceuticals, helping to prevent gastrointestinal (GI) disorders and vaginal infections. In general, kefir is reported to have bacteriostatic effects on Gram-negative bacteria, while being more effective against Gram-positive bacteria.^14^ Due to the health benefits attributed to kefir, its popularity is increasing day by day.^15,16^ In this regard, kefir is associated with a wide range of nutraceutical benefits, including anti-inflammatory, antioxidant, anticancer, antimicrobial, antidiabetic, antihypertensive, and antihypercholesterolemic effects.^15^ Regular consumption of kefir, which is rich in protein, calcium, vitamin B12, vitamin B2, vitamin D, and magnesium, is reported to improve gut health by promoting a healthy microbiota, enhancing antioxidant activity, reducing GI tract infections, and boosting immunity.^17^ Moreover, the microorganisms present in kefir are known to be resistant to low pH and bile acids, to become part of the intestinal microbiota, to inhibit pathogens, and to be beneficial to health.^18^

The lactic and acetic acid bacteria found in kefir, along with the organic compounds formed as a result of fermentation, contribute to its antimicrobial effects. The production method has a significant impact on the quality and characteristics of kefir. In traditional kefir production, kefir grains are used, whereas in commercial production, lyophilized starter cultures are utilized. Although the production methods are similar, the resulting kefirs differ in terms of their sensory, microbiological, chemical, and physical quality characteristics. It is stated that these differences are mostly due to the type of kefir culture used in production.^19–21^ Therefore, it is believed that the antimicrobial properties of kefir produced by using kefir grains and different cultures may also vary.

Antimicrobial activity is one of the key characteristics for evaluating the probiotic potential of a microorganism. The antibacterial activity of probiotics is reported to result from the synthesis of organic acids such as H_2_O_2_, ethanol, phenols, diacetyl, proteins, acetic acid, and lactic acid produced during the growth of probiotics. These metabolites are reported to eliminate and prevent the colonization of pathogens in the body through a competitive exclusion mechanism, whereby probiotics compete with harmful microorganisms for adhesive receptors and nutrients.^22^

Another important factor affecting the quality and product characteristics of kefir, particularly its functional properties, is the presence of prebiotics. Prebiotics are indigestible food components that promote the growth of probiotic microorganisms and positively influence the host by improving gut health.^23^ It is stated that by supporting the development of probiotics, prebiotics also suppress the growth of pathogenic species. Even in the absence of bacteria, prebiotics possess immunomodulatory properties.^24^ Oligosaccharides, which consist of a few monosaccharide units linked by glycosidic bonds, are some of the most well-known prebiotics. They prevent the colonization of pathogens in the intestines and promote their elimination from the body while also supporting the growth of probiotics through the energy they produce. Among the three most recognized prebiotics, galactooligosaccharides stand out from inulin and fructooligosaccharides by not only aiding digestion but also improving the immune system, and by their similarity to human milk oligosaccharides.^25^ Inulin is a carbohydrate that is stable at a pH of around 4–5 and is widely used both as a prebiotic and as a fat replacer.^26^ Products in which probiotic organisms and prebiotic substances are used together, where probiotics selectively utilize the prebiotics and show greater effects together than individually, are defined as synbiotics.^27^ Synbiotics are mixtures that provide health benefits to the host by supporting the development of probiotics in the gastrointestinal system through prebiotic support, enhancing probiotic viability, and promoting their adherence in the colon.^28^ The use of synbiotics is more beneficial than the use of probiotics or prebiotics alone. Studies have shown that various synbiotics, by supporting the growth of probiotic microorganisms in different products, improve the quality characteristics of the final product and enhance antimicrobial effects.^29–32^ The addition of inulin to kefir has been reported to result in higher antimicrobial activity compared to the control, and that increased acidity creates a favorable environment for the growth of probiotic bacteria.^29^

Although various studies have investigated the antimicrobial effects of kefir produced from different types of milk or milk powders (such as whole, semiskimmed, skimmed pasteurized cow, goat, sheep, camel, or buffalo milk),^14,33,34^ as well as microorganisms of different genera and species isolated from kefir [*Lactobacillus*, especially *Lb. kefiri*, *Lactococcus*, *Leuconostoc*, *Acetobacter*, and kefir yeasts (*Kluyveromyces*, *Saccharomyces*, *Torula*)],^35^ there is no detailed research available on the antimicrobial properties of synbiotic kefirs produced from buffalo milk using different methods. Therefore, the aim of the present study is to determine the antimicrobial effects of synbiotic kefirs produced with buffalo milk (with 0.5 and 3.5% fat) enriched with different prebiotics (GOS and inulin), using traditional (grain) and commercial (starter culture) production methods, during storage (on days 1, 7, 14, and 21) against certain pathogenic microorganisms (*Staphylococcus aureus*, *Bacillus subtilis*, *Enterococcus faecalis*, *Listeria monocytogenes*, *Pseudomonas aeruginosa*, *Escherichia coli*, and *Candida albicans*). Additionally, this study aims to contribute to the existing literature on synbiotics, particularly inulin-type fructans, where more information is needed.^36^

## 2 Material and Methods

### 2.1 Materials

The kefir grains used for traditional kefir production were obtained from the Department of Food Engineering, Faculty of Engineering, Süleyman Demirel University (Isparta, Turkey). For commercial kefir production, lyophilized (DVS) cultures were used, which were supplied by Chr. Hansen (Denmark). The mesophilic-thermophilic mixed culture used contained *Debaryomyces hansenii*, *Lactococcus lactis* subsp. *cremoris*, *Lactococcus lactis* subsp. *lactis*, *Lactococcus lactis* subsp. *lactis* biovar *diacetylactis*, *Leuconostoc* spp., and *Streptococcus thermophilus* (FD Direct Vat Set (DVS) eXact Kefir 1). The galactooligosaccharide used as a prebiotic was obtained from Clasado (UK), and the inulin was supplied by Smart Kimya (Izmir, Turkey). The high-density polyethylene (HDPE) packaging material used in the study was obtained from Petek Plastik (Konya, Turkey). The buffalo milk, used as the main raw material in the study, was supplied by a private local dairy farm (Eskişehir, Turkey).

#### 2.1.1 Test Microorganisms

In this study, the following microorganisms were used: *Staphylococcus aureus* (NRRL B-767), *Bacillus subtilis* (wild), *Enterococcus faecalis* (ATCC 29212), *Listeria monocytogenes* (ATCC 7644) (Gram-positive), *Pseudomonas aeruginosa* (ATCC 27853), *Escherichia coli* (ATCC 25922) (Gram-negative), and *Candida albicans* (NRRL Y-12983) (yeast). The results were compared with those of antibacterial control compounds: Levofloxacin, Vancomycin, and the antifungal compound Fluconazole. *E. faecalis* and *E. coli* were obtained from the Faculty of Medicine, Eskişehir Osmangazi University (Eskişehir, Turkey), while *B. subtilis*, *L. monocytogenes*, *S. aureus*, *P. aeruginosa*, and *C. albicans* were obtained from the Department of Biology, Eskişehir Technical University (ESTÜ), Türkiye.

### 2.2 Methods

#### 2.2.1 Kefir Production and Storage

Kefir production was carried out according to Çalışır et al.^21^ According to the method, raw buffalo milk was first preheated (55–60 °C), and then the milk was divided into two portions for fat standardization to achieve fat levels of 0.5% (low-fat) and 3.5% (full-fat). Each group of milk (low-fat and full-fat) was then pasteurized at 95 °C for 5 min and subsequently divided into three subgroups under sterile conditions: galactooligosaccharide (GOS) was added to the first group, and inulin was added to the second group. The third group, to which no prebiotic was added, was considered the control group. The six differently prepared milk groups were cooled to 25 °C, and each milk group was then divided into two: one portion was inoculated with kefir grains (traditional production method), and the other with commercial lyophilized (DVS) culture (commercial production method). As a result of these procedures, a total of 12 different kefir milk formulations (Table 1) were prepared. These were then incubated at 20 °C until the pH dropped to 4.6. Once fermentation was complete, the kefirs were stored in bottles made from high-density polyethylene (HDPE) at 3 ± 1 °C. As detailed above, the experimental design of the study consisted of four factors. The first factor was the fat level (0.5 and 3.5% buffalo milk), the second was the production method (traditional and commercial), the third was prebiotic addition (control, GOS, and inulin), and the fourth factor was storage time (days 1, 7, 14, and 21).

**Table 1:** Synbiotic Kefir Groups Produced Using Buffalo Milk with Different Fat Contents Enriched with Prebiotics

kefir groups	
1	3.5% fat buffalo milk + kefir grains
2	3.5% fat buffalo milk + DVS culture
3	3.5% fat buffalo milk + 2% galactooligosaccharide + kefir grains
4	3.5% fat buffalo milk + 2% galactooligosaccharide + DVS culture
5	3.5% fat buffalo milk + 2% inulin + kefir grains
6	3.5% fat buffalo milk + 2% inulin + DVS culture
7	0.5% fat buffalo milk + kefir grains
8	0.5% fat buffalo milk + DVS culture
9	0.5% fat buffalo milk + 2% galactooligosaccharide + kefir grains
10	0.5% fat buffalo milk + 2% galactooligosaccharide + DVS culture
11	0.5% fat buffalo milk + 2% inulin + kefir grains
12	0.5% fat buffalo milk + 2% inulin + DVS culture

The composition of the raw buffalo milk used for kefir production was as follows: dry matter: 16.81 ± 0.02%, fat-free dry matter: 9.92 ± 0.03%, protein: 4.79 ± 0.05%, fat: 6.88 ± 0.01%, pH: 6.81 ± 0.01, and titratable acidity: 0.14 ± 0.01%.^21^ The fat content of the milk was standardized to 0.5 and 3.5% using a separator. In kefir production, the usage rate of kefir grains was 3%, the usage rate of lyophilized kefir culture was 0.025 g/L, and the amount of prebiotic was determined and added at 2% based on preliminary trials.

#### 2.2.2 Analyses

The antimicrobial and antifungal effects of the 12 different kefir groups produced were determined on days 1, 7, 14, and 21 of storage against certain pathogenic microorganisms (Gram-positive: *S. aureus*, *B. subtilis*, *E. faecalis*, *L. monocytogenes*; Gram-negative: *P. aeruginosa*, *E. coli*; and yeast: *C. albicans*). To determine the antimicrobial activity of the kefir samples, the antibacterial control compounds Levofloxacin and Vancomycin were used, and Fluconazole (1 mg/mL) was used as the antifungal compound.

##### 2.2.2.1 Antimicrobial Activity

The agar well diffusion method was used to observe the antibacterial and antifungal activities of the kefir samples prepared with different formulations. A loopful of freshly cultured microorganisms grown on solid media was inoculated into Mueller-Hinton Broth (MHB) medium and incubated overnight at 37 °C. The resulting cultures were diluted to a concentration of 10^8^ CFU/mL (equivalent to the turbidity of a 0.5 McFarland standard). From each bacterial dilution, 100 μL was pipetted onto the surface of Petri dishes containing 20 mL of Mueller Hinton Agar using a sterile pipet and spread evenly over the medium using a sterile Drigalski spatula. After the plates were allowed to dry, wells with a diameter of 6 mm were made in the agar. Into these wells were injected 50 μL of kefir samples, and positive control solutions (antibacterial control compounds: Levofloxacin, Vancomycin; antifungal compound: Fluconazole [1 mg/mL]) were added. Following overnight incubation at 37 °C, the inhibition zones formed around the wells were measured using a millimeter-scaled ruler.^37^ The results were interpreted based on the diameter of the inhibition zones as follows: + ++: Highly sensitive (≥1,6 cm) + +: Moderately sensitive (1.1–1.5 cm), + : Low sensitivity (0.5–1.0 cm) – : Not sensitive (Inactive, < 0.55 cm).

##### 2.2.2.2 Statistical Analysis

The experiment was designed using a randomized factorial design, considering two milk fat contents (0.5 and 3.5%), three prebiotic types (Control, GOS, and Inulin), two production methods (traditional and commercial), and four storage periods (1, 7, 14, and 21 days). Data were analyzed using mixed model ANOVA (General Linear Model), with factors (fat content, prebiotic type, production method, and storage period) and their interactions as fixed effects and replication as random effects. Statistical analyses were performed in SPSS version 23.0.

## 3 Results and Discussion

### 3.1 Antimicrobial Activity Results of the Control Samples

In recent years, antimicrobial resistance has been increasing and is considered among the most urgent public health concerns. This resistance in microorganisms can arise due to a variety of factors, such as changes in cell membrane permeability, enzymatic modification or inactivation of the antibiotic, modification of the target site, alternative metabolic pathways, and biofilm formation.^38^ In this context, the effects of antibiotics on microorganisms may vary. Within the scope of our study, the antimicrobial activity results of the control compounds Vancomycin, and Levofloxacin against *S. aureus*, *P. aeruginosa*, *B. subtilis*, *E. faecalis*, *E. coli*, *L. monocytogenes*, and Fluconazole against *C. albicans* were presented in Table 2.

**Table 2:** Antimicrobial Activity Results of Control Compounds on Different Microorganism Species (cm)

	control compounds		
tested organism	vancomycin	levofloxacin	fluconazole
Gram-positive bacteria			
*S. aureus*	3.2	2.4	-
*B. subtilis*	3.1	4.2	-
*E. faecalis*	2.8	4.2	-
*L. monocytogenes*	3.2	4.6	-
Gram-negative bacteria			
*P. aeruginosa*	1.8	5.2	-
*E. coli*	3.2	3.8	-
Fungus			
*C. albicans*	-	-	1.8

### 3.2 Antimicrobial Activity Results of Kefir Samples

The antimicrobial activities of the kefir samples were determined against the microorganisms *Staphylococcus aureus*, *Bacillus subtilis*, *Enterococcus faecalis*, *Listeria monocytogenes* (Gram-positive), *Pseudomonas aeruginosa*, *Escherichia coli* (Gram-negative), and *Candida albicans* (yeast). The means and Duncan’s multiple comparison test results regarding the effects of fat content, production method, prebiotic addition, storage time, and their interactions on these microorganisms are presented in Table 3. Although the antimicrobial effects of the kefir samples against the tested microorganisms showed smaller inhibition zones compared with the control compounds, they were found to have antimicrobial activity at varying levels.

**Table 3:** Effect of Fat Levels, Production Methods, Prebiotic and Storage Period on the Antimicrobial Activity of Synbiotic Kefir Samples during Chilled Storage (Inhibition Zone Diameters, cm)^a^

	Gram-positive bacteria				Gram-negative bacteria		Fungus
	*Staphylococcus aureus*	*Bacillus subtilis*	*Enterococcus faecalis*	*Listeria monocytogenes*	*Pseudomonas aeruginosa*	*Escherichia coli*	*Candida albicans*
fat levels (FL)							
0.5%	1.265 ± 0.41	1.175 ± 0.31	1.427 ± 0.64^a^	1.598 ± 0.70^a^	1.215 ± 0.34	1.167 ± 0.40^b^	1.096 ± 0.41
3.5%	1.175 ± 0.28	1.098 ± 0.34	1.256 ± 0.39^b^	1.375 ± 0.37^b^	1.156 ± 0.33	1.350 ± 0.36^a^	1.177 ± 0.36
*P* value	NS	NS	**	**	NS	**	NS
production method (PM)							
traditional	1.133 ± 0.32^b^	1.073 ± 0.30^b^	1.244 ± 0.54^b^	1.473 ± 0.53	1.150 ± 0.30	1.146 ± 0.32^b^	1.056 ± 0.31^b^
commercial	1.306 ± 0.36^a^	1.200 ± 0.34^a^	1.440 ± 0.53^a^	1.500 ± 0.61	1.221 ± 0.36	1.371 ± 0.43^a^	1.217 ± 0.44^a^
*P* value	**	*	**	NS	NS	**	**
prebiotic (P)							
control (C)	1.122 ± 0.23^b^	1.119 ± 0.28	1.409 ± 0.51	1.531 ± 0.56^a^	1.244 ± 0.29	1.172 ± 0.33^b^	0.981 ± 0.27^b^
GOS (G)	1.194 ± 0.38^b^	1.106 ± 0.38	1.300 ± 0.60	1.541 ± 0.68^a^	1.175 ± 0.31	1.375 ± 0.37^a^	1.169 ± 0.29^a^
inulin (I)	1.344 ± 0.38^a^	1.184 ± 0.32	1.316 ± 0.51	1.387 ± 0.44^b^	1.138 ± 0.39	1.228 ± 0.45^ab^	1.259 ± 0.50^a^
*P* value	**	NS	NS	*	NS	*	**
storage period (SP, days)							
1	1.067 ± 0.27^b^	0.971 ± 0.29^c^	1.017 ± 0.34^c^	1.242 ± 0.29^c^	0.983 ± 0.21^c^	1.154 ± 0.35^b^	0.950 ± 0.27^b^
7	1.342 ± 0.27^a^	1.300 ± 0.28^a^	1.300 ± 0.36^b^	1.500 ± 0.30^b^	1.333 ± 0.35^a^	1.433 ± 0.41^a^	1.208 ± 0.24^a^
14	1.100 ± 0.34^b^	1.158 ± 0.34^ab^	1.154 ± 0.40^bc^	1.167 ± 0.24^c^	1.279 ± 0.32^a^	1.267 ± 0.34^ab^	1.129 ± 0.39^a^
21	1.371 ± 0.40^a^	1.117 ± 0.33^bc^	1.896 ± 0.56^a^	2.037 ± 0.78^a^	1.146 ± 0.33^b^	1.179 ± 0.42^b^	1.258 ± 0.51^a^
*P* value	**	**	**	**	**	*	**
interactions							
FL × PM	NS	**	NS	NS	NS	NS	NS
FL × P	**	NS	NS	**	NS	NS	NS
FL × SP	**	**	**	**	**	NS	**
PM × *P*	NS	NS	NS	*	NS	NS	**
PM × SP	NS	NS	*	*	**	NS	**
*P* × SP	**	NS	*	**	**	NS	**
FL × PM × *P*	NS	NS	*	*	*	NS	NS
FL × PM × SP	NS	NS	NS	*	**	NS	NS
FL × *P* × SP	NS	NS	NS	NS	*	NS	NS
PM × *P* × SP	NS	NS	NS	*	**	**	**

a *: *P* < 0.05., **: *P* < 0.01, NS: *P* > 0.05. a-b: Means in the same column and in the same section with the same letters are not significantly different at *P* > *0.05* (Duncan’s test) between the means of the fat levels and production methods. a-c: Means in the same column and in the same section with the same letters are not significantly different at *P* > 0.05 (Duncan’s test) between the means of the prebiotic.

#### 3.2.1 Gram-Positive Bacteria

##### 3.2.1.1 Staphylococcus aureus

The antimicrobial effect of the kefir samples against *S. aureus* was significantly influenced by the production method (*p* < 0.01), the addition of prebiotics (*p* < 0.01), and the storage time. However, the effect of fat content was found to be insignificant (*p* > 0.05). The interactions of fat content × prebiotic addition (*p* < 0.01), fat content × storage time (*p* < 0.01), and prebiotic addition × storage time (*p* < 0.01) on *S. aureus* were found to be highly significant (*p* < 0.01) (Table 3). The protective effect of kefir produced using the commercial method against *S. aureus* was found to be greater than that of the traditionally produced kefir (*p* < 0.05). However, regardless of the production method, kefir samples showed moderate sensitivity (1.1–1.5 cm) with average inhibition zone diameters ranging from 1.133 to 1.306 cm. It was reported that proteolysis of milk by kefir microorganisms during fermentation leads to bioactive peptides with antimicrobial activity. Studies have reported that the mixture of bioactive peptides derived from kefir exhibits antimicrobial activity against various microorganisms, including *S. aureus*.^39^ Among the kefir groups enriched with prebiotics, the group containing inulin (1.344 ± 0.38 cm) was found to most effectively inhibit *S. aureus* growth (*p* < 0.05), while the control group (1.122 ± 0.23 cm) and the group with GOS (1.194 ± 0.38 cm) showed similar effects (*p* > 0.05). This significant difference observed in the inulin-containing group may be attributed to inulin’s support for the production of acids and byproducts in synbiotic samples that inhibit the growth of undesirable microorganisms. Sebayang et al.^29^ reported that the addition of inulin to kefir increased the counts of Gram-positive bacteria, enhanced antimicrobial activity compared to the control, and created a favorable environment for probiotic growth due to increased acidity. During the storage period, the antimicrobial activity of the kefir samples against *S. aureus* did not show a consistent pattern. While the antimicrobial effect increased up to day 7, it decreased on day 14 and increased again by day 21. Throughout the storage period, the kefir samples demonstrated a moderate sensitivity. The variation in antimicrobial activity against *S. aureus* over the storage time was explained by Kim et al.,^40^ who noted that various metabolites and inhibitory compounds found in kefir—, such as organic acids, hydrogen peroxide, ethyl alcohol, diacetyl, peptides, and bacteriocins,—may interact with one another, either enhancing or antagonizing their antimicrobial effects. Therefore, it has been suggested that the antimicrobial activity of kefir may originate from different compounds at each stage of fermentation, which could result in an inconsistent antimicrobial pattern over time. Azizkhani et al.^41^ also stated in their study on kefir and probiotic yogurt produced from different types of milk that differences in antimicrobial activity may arise from numerous parameters, including the composition of fatty acids, the final pH and acidity of the product (which are strongly influenced by lactose content), the types of peptides involved in the production of bioactive compounds, the chemical composition, the type and population of microorganisms, the presence of kefir grains or starter culture, and the diversity of enzymes. The addition of prebiotics to low-fat kefir increased the antimicrobial activity against *S. aureus* compared to the control samples (*p* < 0.05); among the prebiotics, inulin was more effective than GOS in contributing to this increase. However, in high-fat kefirs, no significant difference in antimicrobial activity against *S. aureus* was observed (*p* > 0.05) (Figure 2a). These results indicate that the antimicrobial effect of prebiotics against *S. aureus* changes as the fat content in kefir increases. While GOS did not show variation based on the fat level, inulin enhanced the effect in low-fat kefirs, as clearly shown by the prebiotic × fat level interaction (Figure 1a). During the storage period, the strongest effect was also observed on day 21 in the samples with a 0.5% fat content (Figure 1b). The antimicrobial effect of prebiotics against *S. aureus* varied throughout the storage period. On day 7, kefir with GOS showed the highest effect, while on day 21, kefir with inulin exhibited the strongest antimicrobial activity. By the end of the storage period, no significant difference was observed between the control and GOS groups (*p* > 0.05) (Figure 1c). Al-Mohammadi et al.^42^ reported that kefir produced a 2.1 cm inhibition zone against *S. aureus*. Angelidis et al.^43^ found that the type of kefir grain used in production, the grain ratio, and the initial level of *S. aureus* influenced *S. aureus* growth, and they recommended using a 5% grain ratio to minimize the risk of enterotoxin formation. In line with our research findings, a study by Ender^44^ also showed that kefir produced using fructooligosaccharides (FOS), grains, and starter culture had a greater antimicrobial effect against *S. aureus*, particularly in FOS-added samples produced with starter cultures.

**Figure 1:**
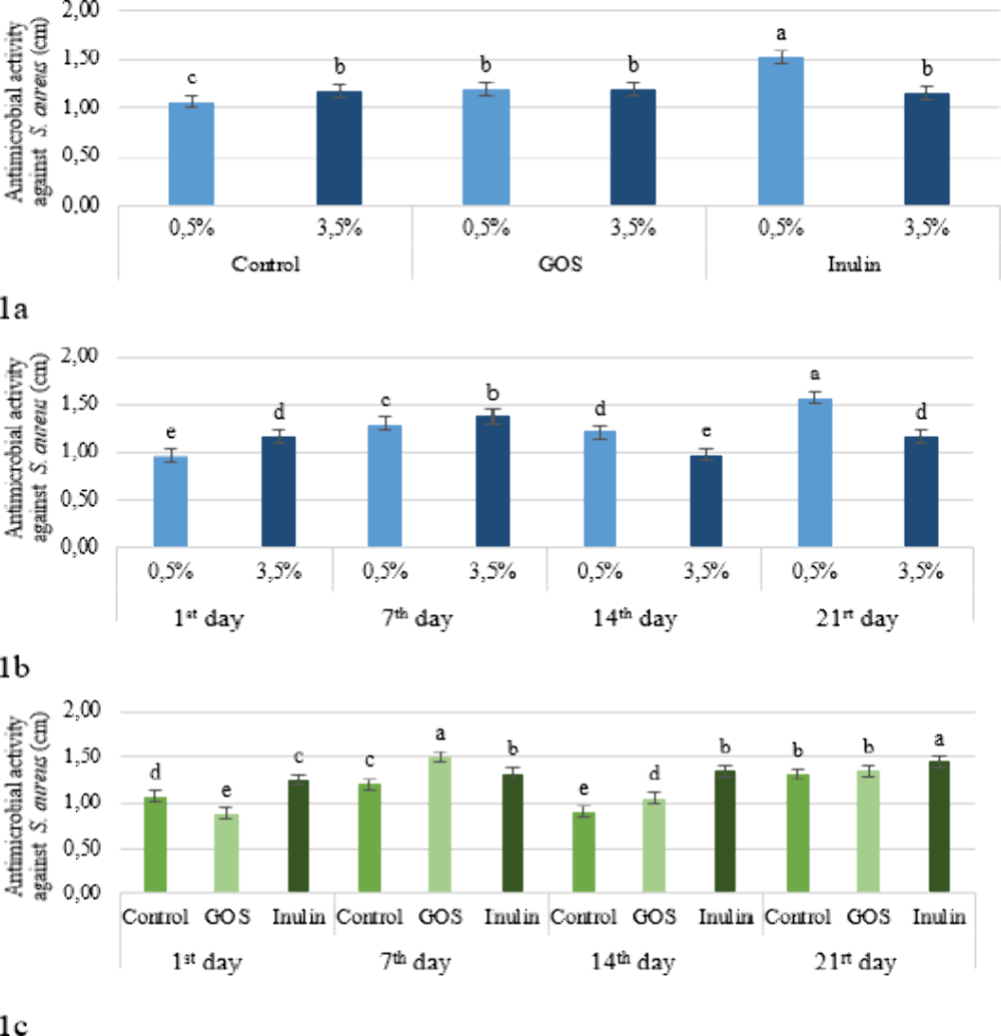
Effect of the fat level × prebiotic (a), fat level × storage period (b), and prebiotic and storage time (c) interaction on the antimicrobial activity against *S. aureus* in the kefir samples (GOS: Galacto-oligosaccharides).

##### 3.2.1.2 Bacillus subtilis

The antimicrobial activity of kefir samples against *B. subtilis* was significantly influenced by the production method (*p* < 0.05) and storage time (*p* < 0.01) (Table 3). The use of starter culture in kefir production-i.e., commercial production method (1.2 cm zone diameter)-resulted in a greater antimicrobial effect against *B. subtilis* compared to the traditional method (*p* < 0.05). On the first day of storage, kefir showed a low level of sensitivity based on the average inhibition zone diameters, whereas on the seventh, 14th, and 21st days, moderate sensitivity was observed (Table 3). Examining the interaction between fat content and the production method (Figure 2a), the 0.5% fat samples showed average inhibition zones of 1.19 cm with grains and 1.16 cm with starter culture, indicating moderate sensitivity. In the 3.5% fat group, grain-based kefir showed an average inhibition zone of 0.95 cm (low sensitivity), while the starter culture group showed 1.24 cm (moderate sensitivity). In low-fat kefirs, the production method had no significant effect on antimicrobial activity, whereas in high-fat kefirs, the commercial method resulted in a greater antimicrobial effect (Figure 2a). In the interaction between fat content and storage time, samples with different fat levels produced varying inhibition zone diameters over the course of storage. The antimicrobial activity against *B. subtilis* increased over the storage period in kefirs with 0.5% fat, whereas in kefirs with 3.5% fat, an increase was observed on day 7, followed by a decrease in the subsequent days (Figure 2b). A previous study also indicated that metabolites formed during kefir fermentation were effective in the inactivation of *B. subtilis*.^39^ No studies have been found specifically regarding the antimicrobial effect of kefir made from buffalo milk on *B. subtilis*. Existing research has focused on different types of milk other than buffalo milk and generally indicates that kefir exhibits antimicrobial activity against this bacterium.^45,46^ On the other hand, lactic acid bacteria, known as a fundamental bacteria of traditional food fermentation processes,^21,47^ are also recognized for their antimicrobial properties, which help prevent and reduce foodborne illnesses by inhibiting pathogenic microbes in food products. These bacteria contribute to food preservation through the production of bacteriocins, hydrogen peroxide, lactic acid, and other organic acids during fermentation. By lowering the pH, they create unfavorable conditions for the growth of pathogens, thereby inhibiting their development. Other organic acids, such as acetic and propionic acids, produced as end-products of fermentation, also exhibit antagonistic effects against bacteria and fungi, even though they are produced in smaller quantities.^48^

**Figure 2:**
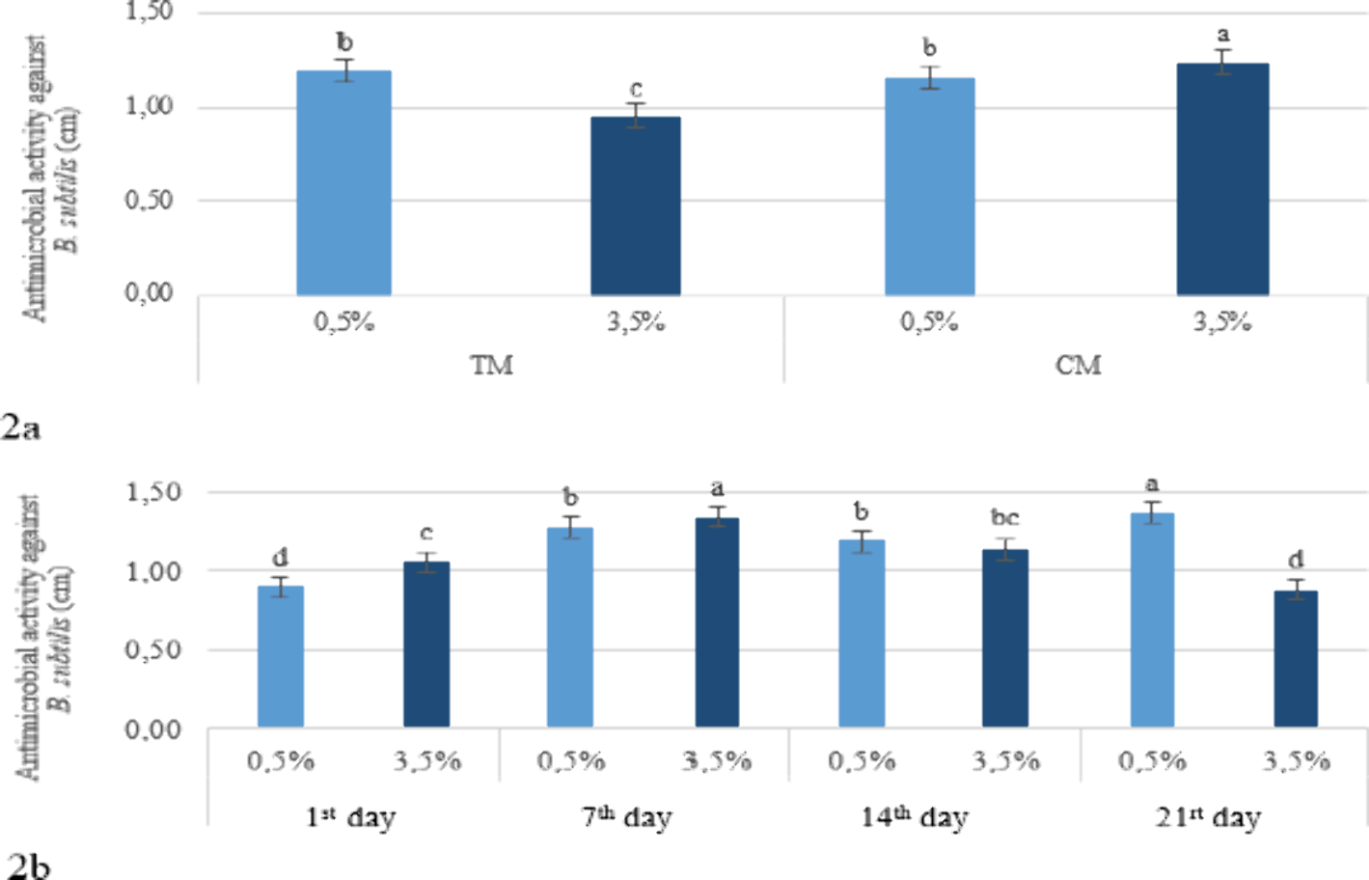
Effect of the fat level × production method (a) and fat level × storage period (b) interaction on the antimicrobial activity against *B. subtilis* in the kefir samples (TM: Traditional Method, CM: Commercial Method).

##### 3.2.1.3 Enterococcus faecalis

*E. faecalis*, as with many processed foods, is an important indicator of fecal contamination, even in heat-treated foods. Therefore, its inactivation by contaminated food components is crucial. In the scope of the study, the kefir samples produced showed varying antimicrobial effects against *E. faecalis*, depending on the treatment applied. The effects of fat level (*p* < 0.01), production method (*p* < 0.01), and storage time (*p* < 0.01) were found to be highly significant (Table 3). In low-fat kefir samples, the antimicrobial activity against *E. faecalis* was stronger than in high-fat samples (*p* < 0.05). This increase was influenced by the fact that the 0.5% fat kefirs showed a high level of sensitivity against *E. faecalis* on day 21, with a zone diameter of 2.37 cm (Figure 3a). In contrast, the 3.5% fat kefirs exhibited moderate sensitivity throughout the storage period (Figure 3a). Products produced using commercial cultures also demonstrated strong activity against *E. faecalis* (Table 3). During the storage period, the sensitivity against *E. faecalis* increased. On day 1 of storage, the average inhibition zone diameter was 1.01 cm, which was classified as low sensitivity (0.55–1.00 cm). On days 7 and 14, the samples were found to have moderate sensitivity (1.1–1.5 cm), and on day 21, they showed high sensitivity (1.6 cm and above) (Table 3). According to the interaction between production method and storage time for the antimicrobial activity against *E. faecalis*, on day 1 of storage, both kefir groups,—those produced with grains and with culture,—showed low sensitivity with average inhibition zone diameters of 1.03 and 1.00 cm, respectively. The kefirs produced with grains showed variability throughout the storage period: 1.20 cm on day 7 (moderate sensitivity), 0.92 cm on day 14 (low sensitivity), and 1.8 cm on day 21 (high sensitivity). Kefirs produced using starter cultures showed moderate sensitivity on days 7 and 14, and high sensitivity on day 21. In general, at the beginning of storage, kefirs produced by both production methods showed similar effects; however, during the later days of storage, a continuous increase was observed, particularly in the samples with added starter culture. On the final day of storage, the highest antimicrobial activity was observed for both production methods (Figure 3b). In the current study, it was determined that the antimicrobial activity against *E. faecalis* was not significantly affected by the addition of prebiotics (*p* > 0.05), whereas the interaction between prebiotic addition and storage time was found to be significant (*p* < 0.05) (Table 3). The highest average inhibition zone diameters observed in prebiotic-containing samples throughout storage were recorded on the 21st day, with kefirs enriched with GOS exhibiting a 2 cm zone (indicating high sensitivity) on that day (Figure 3c). The fact that the lowest antimicrobial activity on the first day of storage was observed in GOS-supplemented kefirs and that by the end of storage this activity had increased to the highest level among all treatments indicates that the antimicrobial effect of GOS against *E. faecalis* increased over the storage period (Figure 3c). Sarhan et al.,^50^ in a study investigating the safety and beneficial properties of kefir grains and strains of the *Enterococcus* genus isolated from kefir, reported that kefir inhibited *E. faecalis*. Similarly, Chifiriuc et al.^45^ demonstrated that kefirs fermented for different durations (24 and 48 h) exhibited antimicrobial activity against *E. faecalis* during a 7-day storage period. Vieira et al.^39^ also reported that the bioactive peptides formed as a result of proteolysis during the fermentation phase of kefir production—mediated by kefir microorganisms—exhibited antimicrobial activity against various microorganisms, including *Pseudomonas aeruginosa*, *Enterococcus faecalis*, *Bacillus subtilis*, and *Staphylococcus aureus*.

**Figure 3:**
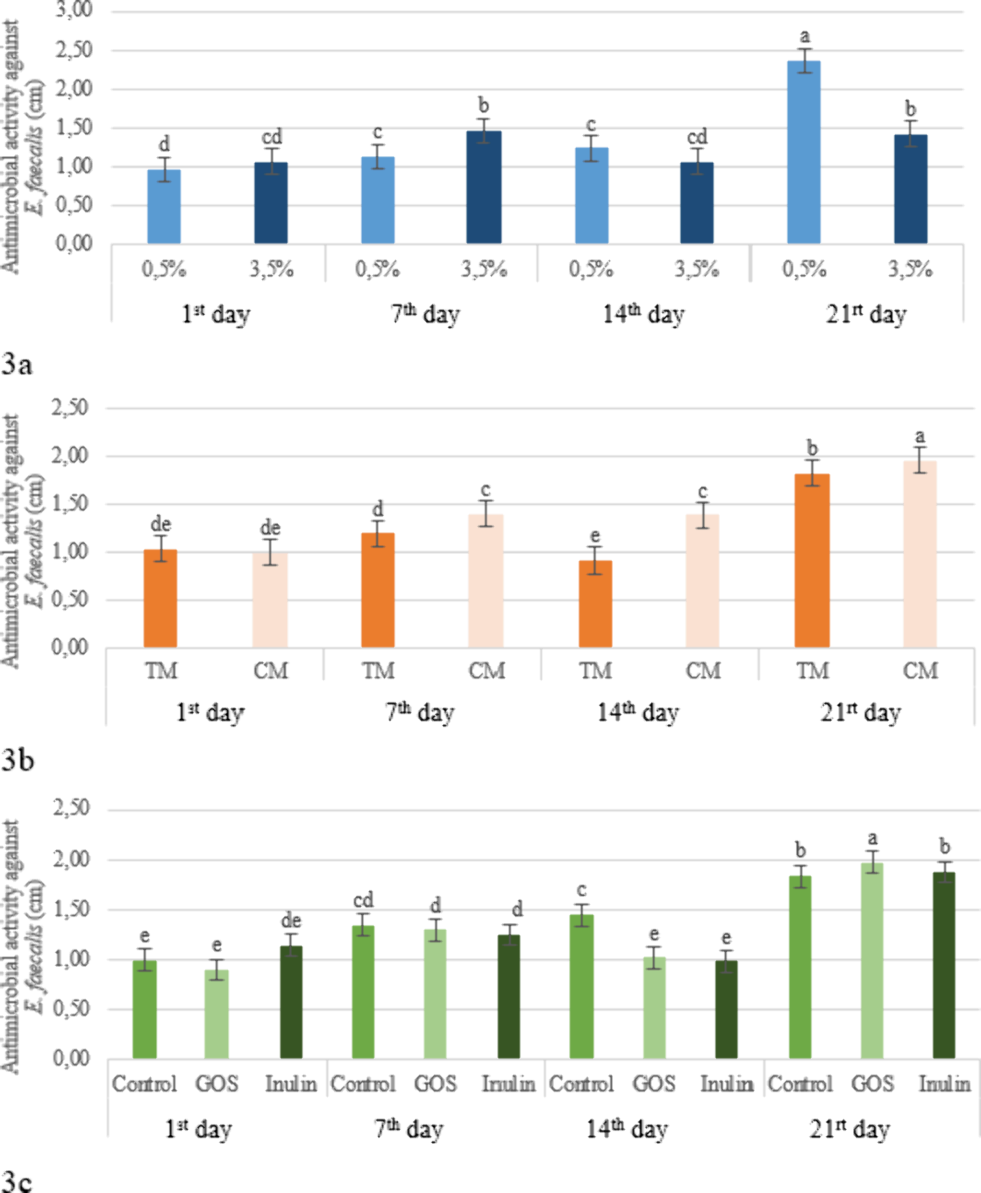
Effect of the fat level × storage period (a), production method × storage period (b), and prebiotic × storage period (c) interaction on the antimicrobial activity against *E. faecalis* in the kefir samples (TM: Traditional Method, CM: Commercial Method, GOS: Galacto-oligosaccharides).

##### 3.2.1.4 Listeria monocytogenes

Changes in the fat content of the milk used in kefir production affected the antimicrobial activity of kefir against *Listeria monocytogenes* (*p* < 0.01), and the antimicrobial effect decreased with the increase in milk fat content (*p* < 0.05). The average inhibition zone diameter created by 0.5% fat kefir was measured at 1.60 cm (highly sensitive), while that of 3.5% fat kefir was 1.38 cm (moderately sensitive) (Table 3). Similarly, in a study conducted by Rugji et al.,^49^ it was reported that milk fat increases the viability of *L. monocytogenes* and that higher fat content has a significant inhibitory effect on pathogen inactivation. The addition of inulin to kefir made from buffalo milk with different fat levels reduced its antimicrobial activity against *L. monocytogenes* (*p* < 0.05), whereas the addition of GOS had no significant effect (*p* > 0.05) (Table 3). As shown in Figure 4a, which presents the fat level × prebiotic interaction, the inhibition zone diameters against *Listeria monocytogenes* were higher in both the control and GOS-supplemented kefirs made with 0.5% fat compared to those made with 3.5% fat. In kefirs supplemented with inulin, however, there was no significant difference between the two fat groups. The results found in our study regarding the effect of GOS are consistent with those reported by Likotrafiti et al.^30^ The researchers observed that the *Lentilactobacillus kefiri* strain isolated from kefir grains grew well in culture media supplemented with the prebiotics FOS, GOS, and lactulose, and that the addition of GOS to the coculture medium significantly inhibited *L. monocytogenes*.^30^ The addition of kefir grains and commercial starter cultures did not affect the antimicrobial activity of kefir against *L. monocytogenes* (*p* > 0.05). However, the interactions between production method × prebiotic (*p* < 0.05, Figure 4b) and production method × storage time (*p* < 0.05, Figure 4d) had significant effects. The addition of GOS enhanced the effectiveness of the traditional method, while the use of inulin increased the effectiveness of the commercial starter culture (Figure 4b). In this context, kefirs produced using a starter culture showed high sensitivity (≥1.6 cm inhibition zone) in the control group, while kefirs produced with grains showed high sensitivity with the addition of GOS. The addition of inulin resulted in moderate sensitivity (1.1–1.5 cm) in both production methods. In both methods, kefirs demonstrated moderate sensitivity during the first 14 days and high sensitivity on day 21 (Figure 4d). Storage time also had a highly significant effect (*p* < 0.01) on the antimicrobial activity of the kefirs against *L. monocytogenes*. On days 1, 7, and 14 of storage, kefirs exhibited moderate sensitivity (1.1–1.5 cm), while on day 21 they showed high sensitivity (≥1.6 cm) (*p* < 0.05) (Table 2). The fact that the inhibition zone formed by low-fat kefirs exceeded 2.5 cm on day 21 (Figure 4c), and that GOS-supplemented products approached this level (Figure 4e), contributed to this increase. Contrary to the current findings, Kalamaki et al.^52^ monitored the growth of *L. monocytogenes* in kefir produced using the traditional method. In their study, they used UHT milk, two different types of grains (at a 5% ratio), and two different storage temperatures (4 and 10 °C). They concluded that the kefir samples were insufficient to inhibit the initially added *L. monocytogenes*, and particularly noted a rapid increase in bacterial count at 10 °C.

**Figure 4:**
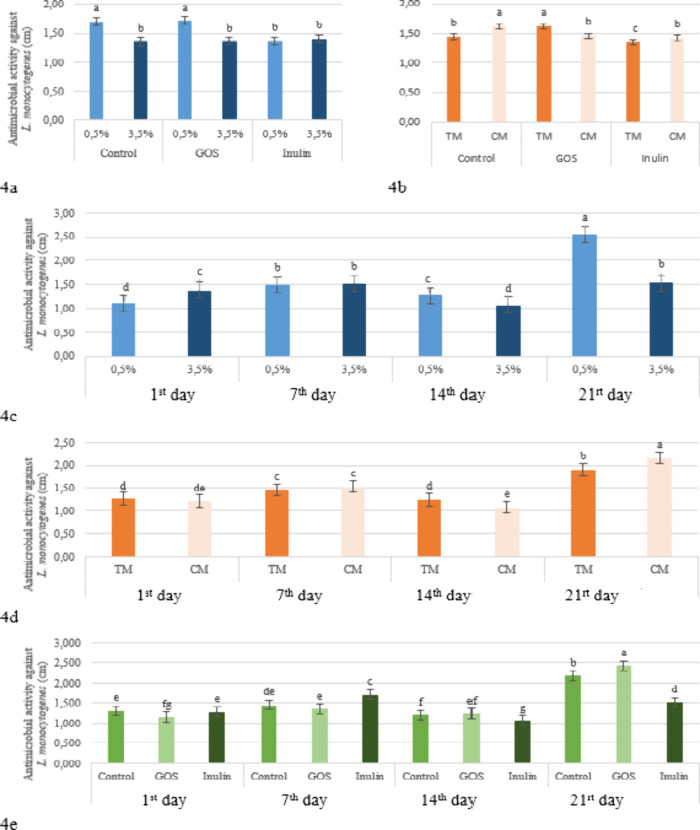
Effect of the fat level × prebiotic (a), production method × prebiotic (b), fat level × storage period (c), production method × storage period (d), and prebiotic × storage period (e) interaction on the antimicrobial activity against *L. monocytogenes* in the kefir samples (TM: Traditional Method, CM: Commercial Method, GOS: Galacto-oligosaccharides).

#### 3.2.2 Gram-Negative Bacteria

##### 3.2.2.1 Pseudomonas aeuroginosa

The fat level of the milk used in kefir production, the production method, and the addition of prebiotics resulted in a moderate sensitivity against *P. aeruginosa*, while the storage period showed low sensitivity on day 1 and moderate sensitivity on days 7, 14, and 21. However, the use of buffalo milk with different fat contents (*P* > 0.05), different production methods (*P* > 0.05), and prebiotic supplementation (*p* > 0.05) did not affect the antimicrobial activity against *P. aeruginosa*, and no statistically significant differences were observed between the samples (Table 3). However, among the factors tested, only the storage period had a highly significant effect (*p* < 0.01) on the antimicrobial activity of the kefir samples against *Pseudomonas aeruginosa*. The interactions between fat level × storage period (*p* < 0.01), production method × storage period (*p* < 0.01), and prebiotic addition × storage period (*p* < 0.01) also significantly influenced the antimicrobial activity against *P. aeruginosa* (Table 3). Except for day 14 of storage, the highest effect was observed in low-fat kefir samples (Figure 5a). At the beginning of storage, no difference was observed between the production methods in terms of inhibition zone formation; however, on days 14 and 21 of storage, kefir produced with commercial cultures was found to be more effective (Figure 5b). The prebiotics added during kefir production showed different effects on the inhibition of *P. aeruginosa* depending on the storage day. On days 1 and 7 of storage, GOS was found to be more effective than inulin, but its effect decreased on days 14 and 21 of storage, during which inulin was more effective. The high count of lactic bacilli in kefir samples produced with GOS on days 1 and 7 of storage increased the effect against *P. aeruginosa*.^21^ Indeed, Sarhan et al.^50^ reported that *Lactobacilli* spp. have a strong antimicrobial effect against *P. aeruginosa*. At the end of 21 days of storage, the effect of GOS was found to be lower than that of the control group, with the order of effectiveness being inulin > control > GOS (Figure 5c). The highest effect against *P. aeruginosa* in kefir samples (approximately 1.5 cm) was observed on the seventh day of storage in both the control and GOS-enriched samples. In a study conducted by Ender,^44^ it was also determined that the storage day had a significant impact on the inhibition of *P. aeruginosa*, with the largest zone diameter observed in kefir samples produced with grains and enriched with FOS. Carasi et al.^51^ reported that kefir grains and strains of the genus *Enterococcus* isolated from kefir increased product safety and beneficial properties, and that many of these strains inhibited pathogens such as *P. aeruginosa*. Vieira et al.^39^ also stated that bioactive peptides formed as a result of proteolysis during kefir production were effective in the inhibition of *P. aeruginosa*.

**Figure 5:**
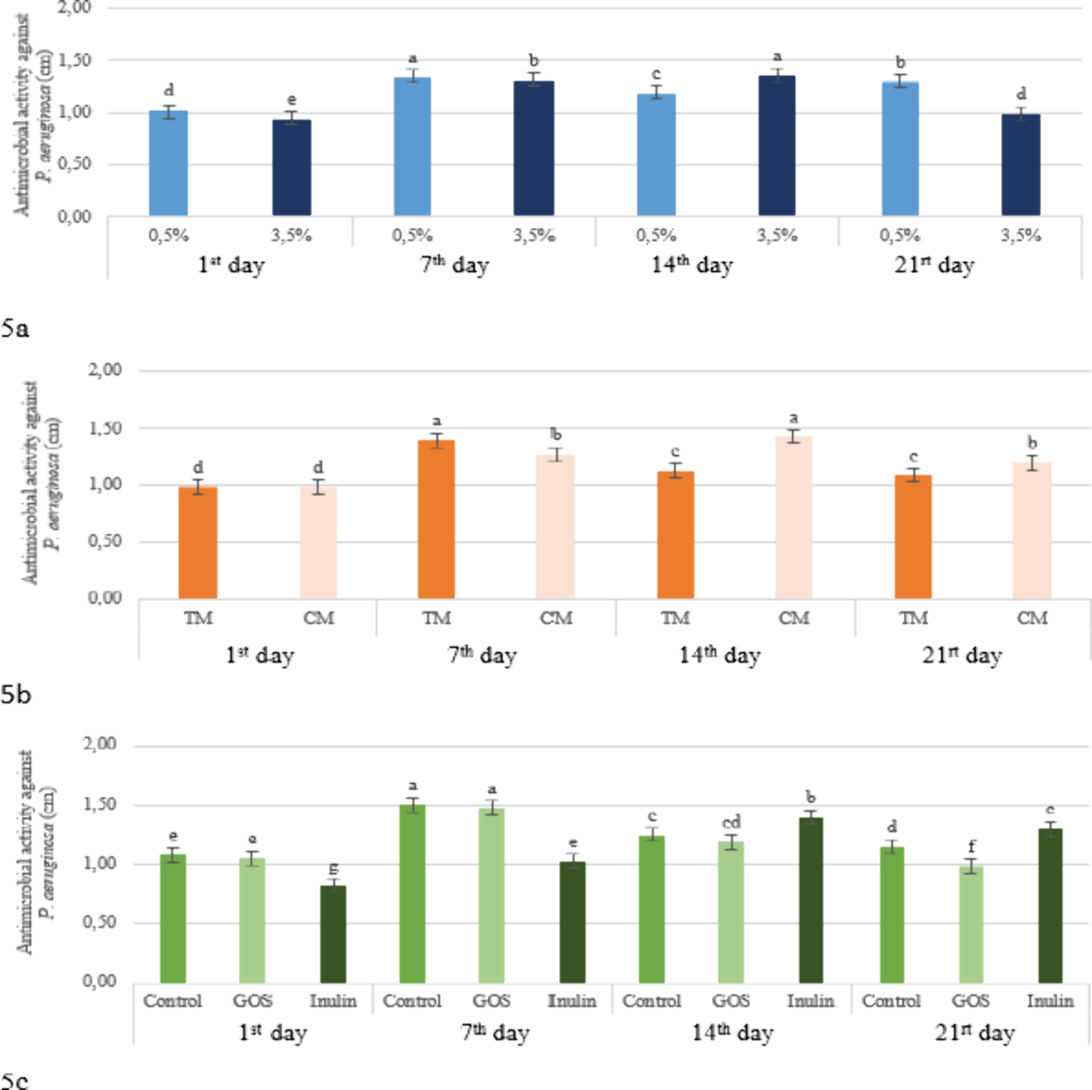
Effect of the fat level × storage period (a), production method × storage time (b) and prebiotic × storage period (c) interactions on the antimicrobial activity against *P. aeruginosa* in the kefir samples (TM: Traditional Method, CM: Commercial Method, GOS: Galacto-oligosaccharides).

##### 3.2.2.2 Escherichia coli

The fat level of the milk used in kefir production had a significant (*p* < 0.01) antimicrobial effect on *E. coli*, and kefirs with 3.5% fat produced larger inhibition zones compared to those with 0.5% fat (*p* < 0.05). In the study, the production method was also found to have a highly significant effect on *E. coli* (*p* < 0.01), with the use of commercial culture resulting in increased inhibition zone diameters (*p* < 0.05). The addition of prebiotics also influenced the antimicrobial activity against *E. coli* (*p* < 0.05), and the highest zone diameter was observed in kefirs supplemented with GOS (*p* < 0.05). The difference in zone size between GOS and the control was approximately 0.2 units. In the present study, the kefirs produced showed varying antimicrobial effects on *E. coli* across different storage days (*p* < 0.05), with the greatest effects occurring on the seventh and 14^th^ days of storage (*p* < 0.05). The difference in inhibition zones between the beginning of storage and day 7 was about 0.3 units, and this increase was mainly influenced by GOS rather than inulin (Table 3). Considering the overall averages, the kefir samples were found to be moderately sensitive (1.1–1.5 cm) to *E. coli* (Table 3). In the study conducted by Hagh,^53^ which investigated the antimicrobial effects of kefir traditionally produced from buffalo milk and grain-type starters, it was reported that buffalo milk kefir exhibited activity against *E. coli*. Ender^44^ stated in his study that the strongest antibacterial effect against *E. coli* was observed in kefirs produced with grains and supplemented with FOS (fructooligosaccharides). Garrote et al.^31^ also found that kefir grains had an inhibitory effect on *E. coli*, attributing this effect to the acetic and lactic acids formed in milk fermented with kefir grains. In their research, Witthuhn et al.^54^ indicated that the antimicrobial effect of kefir was not solely dependent on acidity and pH, but that bioactive compounds such as antimicrobial peptides (bacteriocins) or polysaccharides (exopolysaccharides) could also be effective. In contrast to the existing data, Likotrafiti et al.^30^ reported that the *Lentilactobacillus kefiri* B6 strain they isolated from kefir had no significant effect on *E. coli*

#### 3.2.3 Yeast

##### 3.2.3.1 Candida albicans

*Candida* species are microorganisms capable of utilizing various carbon sources and producing enzymes, acids, and other byproducts.^55^ Recent metagenomic approaches have indicated that *Candida* is present in natural fermentations; however, it is rarely dominant due to the prevalence of other microorganisms and the metabolites and products (such as ethanol and lactic acid) they produce.^56^ Studies indicate that among more than 200 *Candida* species, approximately 20% are considered pathogenic, with *C. albicans* being the most common and invasive species. This species is frequently isolated from hospital environments and is more often considered an opportunistic pathogen in healthy individuals. It is also the species most commonly associated with systemic fungal infections.^36^ In fungal infections, the use of antifungal drugs has been found effective in eliminating infections; however, long-term use of these drugs is reported to lead to the development of resistance in the treatment of fungal infections. Therefore, the use of probiotic-containing products such as kefir, which are among the natural products, is recommended in treatments.^57^ In the present study, the production method (*p* < 0.01), prebiotic addition (*p* < 0.01), and storage time (*p* < 0.01) had highly significant effects on the antimicrobial activity of the kefir samples against *C. albicans*, while the fat content showed no significant effect (*p* > 0.05) (Table 3). In kefirs produced with added cultures, approximately 0.2 cm larger inhibition zones were observed compared to those produced using the traditional method (Table 3). This difference is thought to be due to the commercial cultures being more active and exhibiting greater antifungal effects than grain cultures. Indeed, studies have reported that the microbial composition of kefir can vary depending on its origin, the substrate used during fermentation, and the culturing methods. It has been noted that the microorganisms in kefir grains produce lactic acid, antibiotics, and bactericides that inhibit the growth of spoilage and pathogenic microorganisms in kefir milk.^58^ With the addition of prebiotics, the inhibition of *C. albicans* increased, and although the difference was not statistically significant, inulin showed a 0.1 cm advantage over GOS. Prebiotics are indigestible food components that selectively stimulate the growth and/or activity of probiotics, thereby providing beneficial effects to the host. Commonly used prebiotics include inulin, fructooligosaccharides (FOS), galactooligosaccharides (GOS), soy oligosaccharides, xylooligosaccharides, pyrodextrins, isomaltooligosaccharides, and lactulose.^59^ In the study conducted by Çalışır et al.,^21^ it was reported that acidity increased more in kefirs produced from 3.5% fat buffalo milk with added inulin compared to the control and GOS groups. Similarly, Aktaş et al.^47^ found that in concentrated kefirs produced from buffalo milk, the acidity ranged between 1.07 ± 0.03 and 1.16 ± 0.02% during a 28-day storage period. At the beginning of storage, the average inhibition zone diameter was 0.950 cm, which increased after the seventh day of storage, with no statistically significant difference observed between days 7 and 21 (*p* > 0.05). The difference in zone diameter between days 1 and 21 of storage was approximately 0.310 units, which is considered a significant difference. This change may be attributed particularly to the fermentation products formed from the beginning of storage and the prebiotics used. Studies have indicated that prebiotics have a protective effect during product storage by enhancing the survival and activity of selected and dominant probiotics.^60,61^ Mazloomi et al.^62^ reported that the addition of inulin (1 and 2%) to milk increased the viability of yogurt bacteria during the storage of synbiotic yogurt. When examining the fat content × storage time interaction, which was found to be highly significant (*p* < 0.01) in terms of antimicrobial activity against *C. albicans* (Figure 6a), notable differences were observed between storage days and fat levels. The lowest average activity values were detected at the beginning of storage, while no significant differences were found between the averages on other storage days (*p* > 0.05) (Table 3). However, significant differences were observed between fat levels on each storage day. Kefir samples with 3.5% fat content exhibited antifungal activity against *C. albicans* on all storage days except day 21. In 3.5% fat kefirs, the inhibition zone diameter increased up to day 14 and then slightly decreased, reaching 1.19 cm, indicating moderate sensitivity. Flavoring compounds such as diacetyl and menthol, naturally present in milk fat or added to it, enhance antifungal activity. Menthol, in particular, inhibits *C. albicans*. However, in the current study, low-fat (0.5%) kefirs reached the largest average inhibition zone (1.33 cm) against *C. albicans* on day 21 of storage. The type of milk used in kefir production also influences its inhibitory effect against *C. albicans*. In a study conducted by Azizkhania et al.,^41^ the highest inhibitory effect against *C. albicans* was found in sheep milk kefir, followed by kefirs made from camel, goat, and cow milk. However, no studies have been found regarding buffalo milk. The addition of prebiotics to kefirs produced using the commercial method increased activity against *C. albicans*, whereas the addition of prebiotics in the traditional method showed no effect (Figure 6b). In the traditional method, the largest inhibition zone diameter was observed in the samples with GOS, with an average of 1.1 cm (moderate sensitivity). In the commercial method, the highest average diameter was recorded with inulin use, at 1.46 cm (moderate sensitivity). These results indicate that the use of prebiotics in kefir production by the commercial method enhances the inhibition of *C. albicans*, and the contribution of inulin in this effect is greater than that of GOS. The beneficial effects of probiotics may be related to the type and dose of prebiotics used. Due to its selective fermentation nature, inulin has the ability to alter the composition of the microflora by increasing the counts of bacteria that can promote health and potentially reduce harmful bacteria. In this context, inulin is reported to inhibit enteropathogenic bacteria and stimulate the growth and activity of beneficial microorganisms. When fermented by beneficial bacteria, inulin leads to a decrease in pH and the production of acids, including short-chain fatty acids, which are effective in inhibiting pathogens.^36^ In a study by Çalışır et al.,^21^ it was also found that the pH value of inulin-added kefirs (4.50 ± 0.12) produced from buffalo milk was lower than that of GOS-added samples (4.56 ± 0.12). The researchers also determined that the yeast count was approximately 1.5 logarithmic units lower in kefirs produced with commercial culture and inulin compared to those produced with GOS. According to the production method × storage time interaction for antimicrobial activity against *C. albicans*, all groups showed the lowest activity on the first day of storage (0.55–1 cm). In the traditional method, the inhibition zone diameter increased until day 7 and then decreased after day 14. In the commercial method, values were higher on days 7 and 14 compared to other storage days. On day 14, no difference was observed between the production methods, while on the other days, the inhibition zones were larger in the culture-added samples than in those produced with grains. The largest inhibition zone diameter with grain use was observed on day 14, while with culture use, it was seen on day 21 (Figure 6c). Both results indicate moderate sensitivity to *C. albicans*. The production method affects product quality and storage durations. In this study, the use of commercial culture had a greater impact than grains in inhibiting *C. albicans*, which is considered a pathogenic yeast. It is emphasized that probiotics must remain viable throughout the entire shelf life of the product. However, the viability of probiotics in commercial preparations is affected by various factors such as temperature, acidity, the presence of other microorganisms, and oxygen. Therefore, inulin or oligofructose is frequently used in studies aimed at improving bacterial viability.^61^ As shown in the prebiotic addition × storage time interaction for antimicrobial activity against *C. albicans* (Figure 6d), the inhibition zone diameters of prebiotic-enriched kefirs were higher than those of the control throughout the storage period. Although there was no difference between GOS and inulin on day 14 of storage, inulin was more effective on day 21. In a study where inulin and certain fruits were used as prebiotics,^63^ it was found that prebiotic supplementation increased beneficial metabolites against *Candida* growth, and it was suggested that probiotics and prebiotic supplementation could be an effective alternative for *Candida* infections. In another study investigating the effects of inulin and galactooligosaccharides (GOS), along with certain other prebiotics, on fruit juice fermentation (72 h), it was reported that inulin more significantly enhanced the proliferation of *L. plantarum* compared to GOS and control lactose. It was noted that chain length plays an important role in determining which species can ferment a specific prebiotic, and due to its chain length, inulin was more effective than GOS and lactose.^64^

**Figure 6:**
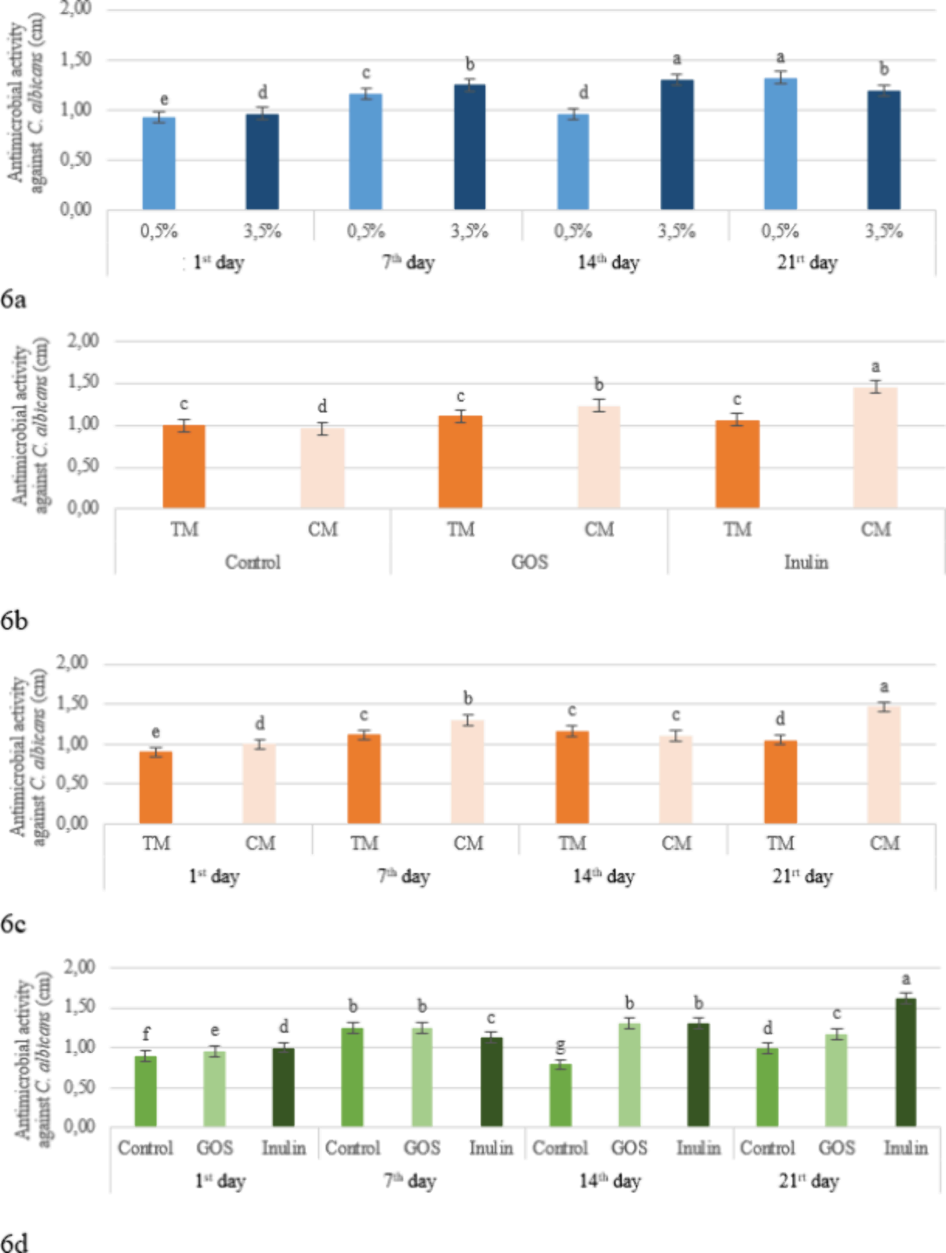
Effect of the fat level × storage period (a), production method × prebiotic (b), production method × storage period (c), and prebiotic × storage period (d) interaction on the antimicrobial activity against *C. albicans* in the kefir samples (TM: Traditional Method, CM: Commercial Method, GOS: Galacto-oligosaccharides).

## 4 Conclusion

The antimicrobial effects of synbiotic kefirs produced with buffalo milk against *S. aureus*, *P. aeruginosa*, *B. subtilis*, *E. faecalis*, *E. coli*, *L. monocytogenes*, and *C. albicans* varied depending on the treatment applied. In general, the use of cultures in fermentation was more effective compared with the use of grains. The addition of prebiotics (GOS, inulin) to buffalo milk increased antimicrobial activity against *E. coli* and *C. albicans*. Inulin addition enhanced antimicrobial activity against *S. aureus* but decreased it against *L. monocytogenes*. In the study, it was found that as the fat content of the buffalo milk used in kefir production increased, antimicrobial activity against *E. faecalis* and *L. monocytogenes* decreased, while it increased against *E. coli*. Overall, the antimicrobial activity of the kefirs produced increased with longer storage periods. In conclusion, the present study determined that the synbiotic kefir samples produced with buffalo milk and prebiotic supplementation exhibited antibacterial and antifungal effects; however, the inhibition zones observed were smaller than those formed by the control compounds. According to the results of this study, the kefir produced from buffalo milk has the potential to contribute positively to supportive therapy against foodborne pathogens.
